# Data on photovoltaic system using different perturb and observe methods under fast multi-changing solar irradiances

**DOI:** 10.1016/j.dib.2017.12.048

**Published:** 2018-01-03

**Authors:** Lele Peng, Shubin Zheng, Wei Xu, Li Xin

**Affiliations:** School of Urban Railway Transportation, Shanghai University of Engineering Science, No. 333 Longteng Road, Songjiang, Shanghai 201620, China

## Abstract

This article presents the data on photovoltaic (PV) system used different perturb and observe (P&O) methods under fast multi-changing solar irradiances. The mathematical modeling of the PV system and tangent error P&O method was discussed in our previous study entitled “A novel tangent error maximum power point tracking algorithm for photovoltaic system under fast multi-changing solar irradiances” by Peng et al. (2018) [1]. The data provided in this paper can be used directly without having to spend weeks to simulate the output performance. In addition, it is easy to apply the results for comparison with other algorithms (Kollimalla et al., 2014; Belkaid et al., 2016; Chenchen et al., 2015; Jubaer and Zainal, 2015) [2,3,^4^,5], and develop a new method for practical application.

**Specifications Table**

**Table:** 

Subject area	Energy
More specific subject area	Solar energy
Type of data	Table
How data was acquired	The values of the materials have been accumulated from the Refs. [2], [3]. Afterwards, the simulation was conducted by the tool of Matlab/Simulink. And the experimental results was carried out by Boost converter.
Data format	filtered, analyzed
Experimental factors	A proper initial value and perturbation step are calculated by the model of PV system.
Experimental features	The dynamic performance of PV system with high efficiency is obtained by different P&O methods.
Data source location	Shanghai University of Engineering Science, Shanghai 201620, China
Data accessibility	Data is provided with this article

**Value of the data**

• The data presented in this paper can save time of other researchers who may need to apply this data for comparison with other algorithms.
• Using these dataset, researchers can easily develop a high efficiency method for practical application.
• These data will helpful as they can be available for testing new algorithms and their performance may be measured by the use of these data.

## 1 Data

This paper presents the numerical data for PV system based on Boost converter under fast multi-changing solar irradiances. The simulation was carried out by the tool of Matlab/Simulink on a Core i5 computer with Win7 operating system. By using ARM controller STM32F103, the experimental platform of prototype PV system was built up. And the specifications for the PV system are shown in Table 1.

**Table 1:** Specifications for the PV system.

MSX60 PV module (*T*_*ref*_ = 25 °C,*G*_*ref*_ = 1000 W/m^2^, AM1.5)	
Output rating power, $P_{\max\;,ref}$ (W)	60
Open circuit voltage, $V_{oc,ref}$ (V)	21.1
Short circuit current, $I_{sc,ref}$ (I)	3.8
Voltage at the maximum power point, $V_{mp,ref}$ (V)	17.1
Current at the maximum power point, $I_{mp,ref}$ (I)	3.5
Temperature coefficient for short circuit current, $K_{i,sc}$ (A/K)	0.003
Temperature coefficient for open circuit voltage, $K_{v,oc}$ (V/K)	−0.0073
Number of PV cell in series, $n_{s}$	36
Number of PV cell in parallel, $m_{p}$	2

Boost converter	
Inductance, $L_{1}$ (mH)	2
Input capacitor, $\left(C_{1}(\mu F)\right)$	470
Output capacitor, $C_{2}\;(\mu F)$	1000
Switching frequency, f (kHz)	20

MPPT controller	
Proportional constant, P	0.5
Integral constant, I	0.125
Initial voltage, $V_{pv}(0)$ (V)	16.79
Initial step size, $\Delta V_{pv}(0)$ (V)	0.0012
Threshold value, K(W/V)	0.14

## 2 Experimental design, materials, and methods

To obtain the output results of PV system under fast multi-changing solar irradiances, a dynamic irradiance profile is given, which is done for duration of 2.6 s at 25 °C temperature [4]. By using the conventional, Ref. [5] and tangent error P&O methods, Tables 1–4 of Appendix A show the output results, respectively.
