# Supplementary material for: Data on photovoltaic system using different perturb and observe methods under fast multi-changing solar irradiances
Source: Data Brief. 2018 Jan 3;17:169–71. doi: 10.1016/j.dib.2017.12.048 (PMC5988016; doi:10.1016/j.dib.2017.12.048)
Supplement: Supplementary file 1 — Supplementary material [file mmc1.doc]

**CONFLICT OF INTEREST STATEMENT**

**Title: Data on photovoltaic system using different perturb and observe methods under fast multi-changing solar irradiances.**

We, the authors of this data article, state that we don’t have any conflict of interest with any researcher, editorial member, reviewer, organization, etc.

Regards,

Lele Peng (corresponding author)

Email: [peter.peng_01@139.com](mailto:peter.peng_01@139.com)
